# Proximal seminal vesicle displacement and margins for prostate cancer radiotherapy

**DOI:** 10.1002/jmrs.457

**Published:** 2021-01-12

**Authors:** Daryl Lim Joon, Michael Chao, Angelina Piccolo, Michal Schneider, Nigel Anderson, Monica Handley, Margaret Benci, Wee Loon Ong, Karen Daly, Rebecca Morrell, Kenneth Wan, Nathan Lawrentschuk, Farshad Foroudi, Trish Jenkins, David Angus, Morikatsu Wada, Shomik Sengupta, Vincent Khoo

**Affiliations:** ^1^ Department of Radiation Oncology Olivia Newton‐John Cancer Wellness and Research Centre Austin Health Melbourne Vic. Australia; ^2^ Monash University Melbourne Vic. Australia; ^3^ Department of Urology Austin Health Melbourne Vic. Australia; ^4^ Royal Marsden NHS Foundation Trust London UK

**Keywords:** Clinical target volume, displacement, fiducial markers, margins, planning target volume, prostate cancer, radiotherapy, radiotherapy planning, seminal vesicles

## Abstract

**Introduction:**

Guidelines recommend that the proximal seminal vesicles (PrSV) should be included in the clinical target volume for locally advanced prostate cancer patients undergoing radiotherapy. Verification and margins for the prostate may not necessarily account for PrSV displacement. The purpose was to determine the inter‐fraction displacement of the PrSV relative to the prostate during radiotherapy.

**Methods:**

Fiducials were inserted into the prostate, and right and left PrSV (RSV and LSV) in 30 prostate cancer patients. Correctional shifts for the prostate, right and left PrSV and pelvic bones were determined from each patient's 39 daily orthogonal portal images relative to reference digitally reconstructed radiographs.

**Results:**

There was a significant displacement of the RSV relative to the prostate in all directions: on average 0.38 mm (95% confidence interval (CI) 0.26 to 0.50) to the left, 0.80–0.81 mm (CI 0.68 to 0.93) superiorly and 1.51 mm (CI 1.36 to 1.65) posteriorly. The LSV was significantly displaced superiorly to the prostate 1.09–1.13 mm (CI 0.97 to 1.25) and posteriorly 1.81 mm (CI 1.67 to 1.96), but not laterally (mean 0.06, CI −0.06 to 0.18). The calculated PTV margins (left–right, superior–inferior, posterior–anterior) were 4.9, 5.3–5.6 and 4.8 mm for the prostate, 5.2, 7.1–8.0 and 9.7 mm for the RSV, and 7.2, 7.5–7.6 and 8.6 mm for the LSV.

**Conclusion:**

There is a significant displacement of the PrSV relative to the prostate during radiotherapy. Greater margins are recommended for the PrSV compared to the prostate.

## Graphical abstract

Seminal vesicles displacement is greater than that of the prostate during radiotherapy and requires a greater margin.

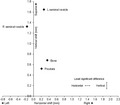

## Introduction

Seminal vesicle invasion (SVI) occurs in 7–24% of prostate cancer patients at presentation.^1^ It is an important poor prognostic factor, indicative of aggressive disease with a high risk of metastases.^1^ However, it is not uniformly fatal.^2^ Radiotherapy studies have shown an outcome improvement of high‐risk patients, including SVI, with dose escalation^3^ or androgen deprivation therapy.^4^ The SWOG 8794 subset analysis of post‐prostatectomy patients with SVI showed that adjuvant radiotherapy led to a significantly improved recurrence‐free survival and a trend to better overall survival.^5^

The risk for SVI in prostate cancer can be estimated using either Partins Tables or Roach's formulae.^6^ It is generally recommended that the seminal vesicle (SV) be included in the clinical target volume (CTV) for intermediate to high and very high‐risk categories.^6^, ^7^ Inclusion of the SV in the CTV is important so that the SV receives an adequate dose.^8^

Daily online targeting is verified according to the prostate position and does not necessarily account for SV displacement. Studies have illustrated that the SV can move in relation to the prostate.^9^, ^10^, ^11^, ^12^, ^13^, ^14^, ^15^ Most analysed the motion of the whole SV to the tips. However, prostatectomy pathological analysis has shown that the SVI rarely extends beyond the proximal 2.0–2.5 cm.^16^ Guidelines recommend that only the proximal 1–2 cm seminal vesicles (PrSV) should be included in the CTV.^16^ Measures of the whole SV motion and margins may not accurately reflect the PrSV.

The present study has minimised observer uncertainties by inserting gold fiducial markers both the prostate and PrSV. This exploratory study aimed to quantify the inter‐fraction PrSV displacement relative to the prostate and evaluate the related planning target volume (PTV) margins of the PrSV as recommended in clinical guidelines.

## Methods

### Study cohort

This study was approved by Austin Health Human Research Ethics Committee. The overriding eligibility criteria were men with locally advanced prostate cancer,^7^ where the inclusion of the SV in the radiotherapy volume was indicated. Patients were recruited prospectively after signing informed consent. The planned sample size of 25 men was considered sufficient to estimate margins. Some patients were excluded because of incorrectly placed fiducials, and therefore, the protocol was amended and approved by ethics to increase the patient accrual number.

### Gold seed insertion technique

The Northwest Medical Physics Equipment (NMPE) ® Soft Tissue Marker Kit (P/N 887‐825) was utilised. Three 3 mm × 1.2 mm gold fiducials were inserted by a single experienced urologist under sedation and antibiotic prophylaxis using trans‐rectal ultrasound guidance. The SV fiducials were inserted to define the proximal 2 cm of the SV and confirmed at CT simulation. The five fiducials were positioned as follows: Seed 1 on right prostate base, Seed 2 on left prostate mid‐gland, Seed 3 on the right prostate apex, Seed 4 on right seminal vesicle (RSV) and Seed 5 on left seminal vesicle (LSV).

### Image acquisition

CT simulation was performed on a General Electric Radiation Therapy Lightspeed Widebore ® helical scanner (General Electric Healthcare, Buckinghamshire, United Kingdom) with a resolution of 512 × 512 pixels, pitch 0.75, no gap and a slice thickness of 1.25 mm, two weeks following fiducial insertion. Patients were positioned supine with a custom foam Alpha cradle placed on an indexed pelvic board with foot stocks. A standard bladder and bowel protocol was used to have a comfortably full bladder and empty rectum (Microlax enema). Orthogonal digitally reconstructed radiographs (DRRs) were generated from the CT and used as the reference images for verification. All were treated to a total dose of 78 Gy in 39 fractions using the departmental intensity‐modulated radiotherapy (IMRT) protocol on an Elekta ® Synergy linear accelerator (Elekta, Stockholm, Sweden). Daily pre‐treatment orthogonal electronic megavoltage portal verification images were performed for each 39 fractions, that is an anterior–posterior image (API) and left lateral image (LLI) with a resolution of 512 x 512 pixels.

### Image verification

Two trained observers (radiation therapists) independently matched the daily verification images with the reference DRRs using Elekta iView® software (Elekta, Stockholm, Sweden) using four different marker matching methods relative to the initial setup. The four matching methods were prostate three seeds (prostate), RSV seed, LSV seed and pelvic bones (Bone) for historical comparison. The observers were blinded to the other observer's matches. The correctional shifts were recorded for each of the four‐different marker matches in millimetres for the two images – API and LLI, that is

1. Lateral left–right (LR) and superior–inferior (SI) correctional shifts for the API and
2. Anterior–posterior (AP) and SI shifts for the LLI.

### Statistical methods

The statistician independently checked all the data for data entry errors by comparison with the original handwritten records. No genuine outliers were excluded.

To compare the fiducials, analysis of variance (ANOVA) was performed on the API and LLI separately, adjusting for patients, fractions within patients and observers. If the ANOVA showed significant differences between fiducials (*P* < 0.05), their means were compared using t‐tests based on the ANOVA standard error of the difference. As there were no pre‐specified hypotheses and six possible pairwise comparisons between the four fiducials, the least significant difference between the means at the 5% level (LSD_0.05_) was adjusted for the number of non‐significant comparisons, if any, using the Hochberg–Benjamini modification of the Bonferroni correction to maintain the overall probability of a false positive conclusion (Type 1 error) at less than 0.05.^17^ Ninety‐five per cent confidence intervals (CI) for differences between means were calculated as the difference ± LSD_0.05_.

To assess the adequacy of PTV margins, the means and standard deviations (SD) for each patient over 39 fractions and two observers were calculated. We then derived the overall mean (group systematic error), the SD of the means (systematic error, Σ) and the root mean square of the SD (random error, σ). The margins were calculated according to the formula: Margin = 2.5Σ + 0.7σ.^18^ The PTV margins calculated from the inter‐fraction displacements take into account uncertainties including setup, delineation/verification and inter‐fraction motion. The formula does not account for the overall mean shift (group systematic error) of the seminal vesicles relative to the prostate. GenStat statistical software, version 18.1, was used for the analysis.

## Results

### Baseline characteristics of the study cohort

The patients were accrued over three years from 7 August 2006 to 15 May 2009. Forty‐three patients were enrolled, but 13 were excluded from analysis because at least one seed was incorrectly placed, that is missing, not within the prostate or not in PrSV (eight), had migrated (one), or images were unclear or lost due to power failure (four). The final cohort consisted of 30 men with locally advanced prostate cancer, that is two patients (7%) with NCCN^7^ very high risk, 13 (43%) with high risk and 15 (50%) with intermediate‐risk prostate cancer. The mean age was 69 years (SD 6.3, range 55–77). The mean PSA was 12 (SD 10.3, range 0.4–54.2). T stages were T1c (*n* = 10), T2 (*n* = 10) and T3 (*n* = 10). All but three had Gleason scores ≥ 7.

The total data set potentially consisted of 18,720 shifts in two dimensions (30 patients × 39 fractions × 2 images × 2 observers × 4 marker sites). Of these, 190 shifts (1%) were missing. The reasons were image problems: image not taken, lost or poor quality (132 shifts or 0.7%) or observer errors, for example measured the wrong image or not appropriately recorded (58 shifts or 0.3%).

### Comparison of fiducials on the anterior–posterior image

**Figure 1:**
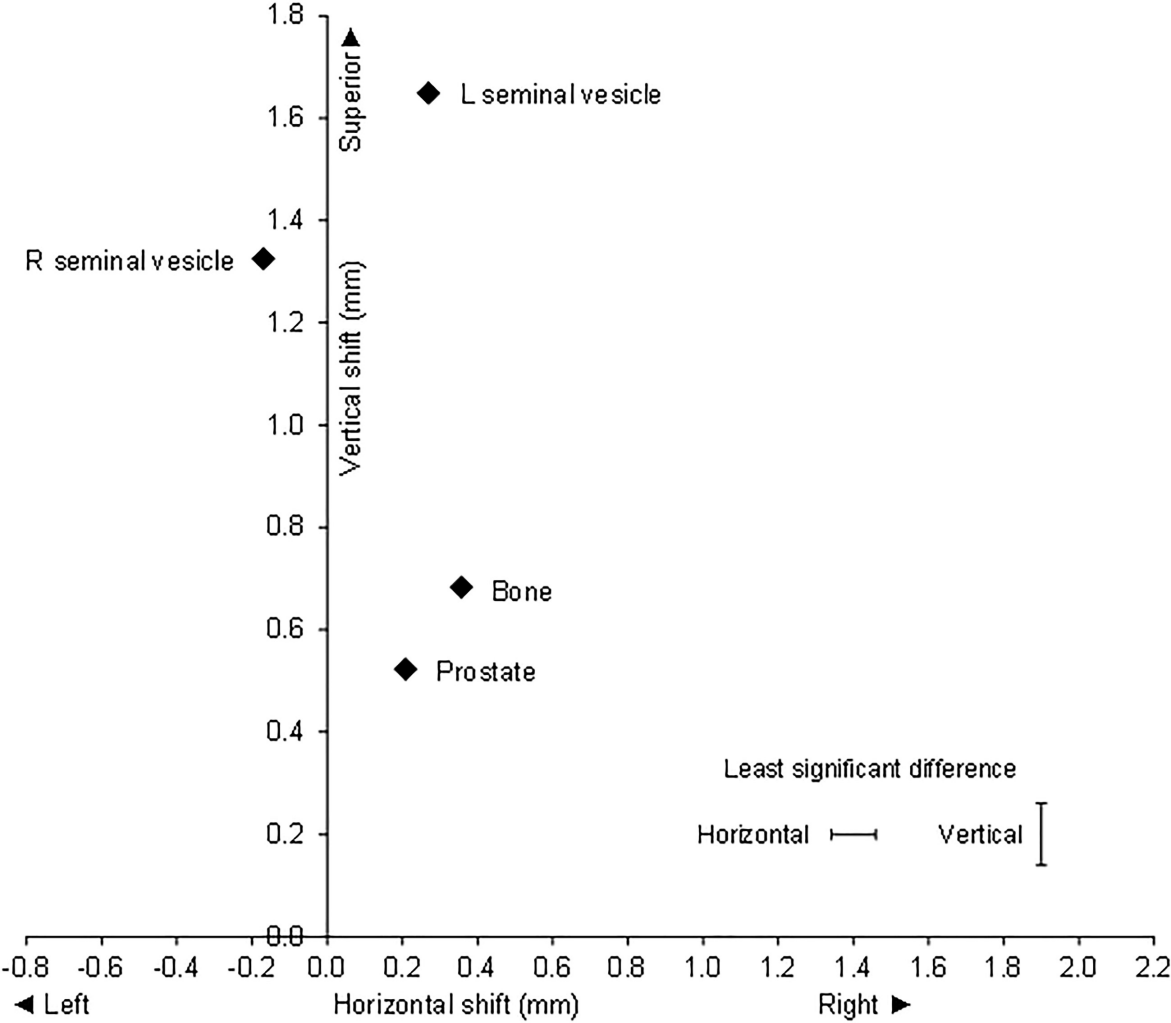
Anterior–posterior image: Mean correctional verification shifts with respect to bone and gold seed fiducial in prostate, right seminal vesicle and left seminal vesicle.

The ANOVA on the shifts for the AP image showed there were highly significant differences between fiducials, both in the LR and SI directions (*P* < 0.001, *n* = 9250) (Fig. 1). Overall, the prostate, bone and left seminal vesicle shifted to the right, and the right seminal vesicle shifted to the left compared to the initial setup. All fiducials shifted superiorly, with the LSV moving the most.

On average, the RSV and LSV correctional shifts were significantly greater than the prostate in the superior direction, and the RSV shifts were significantly more to the left than the prostate or the LSV. The mean shifts in mm for each verification method (LR, SI) were prostate (0.21, 0.52), RSV (−0.17, 1.33), LSV (0.27, 1.65) and bone (0.36, 0.68). The standard errors of the difference between means for LR and SI directions were 0.050 and 0.062 mm, respectively. The least significant differences (*P* < 0.05) were 0.12 for the LR direction (adjusted for 2 non‐significant comparisons) and 0.12 mm for the SI direction (no adjustment required).

The displacement of the RSV relative to the prostate was 0.38 mm to the left (CI 0.26 to 0.50) and 0.80 mm superiorly (CI 0.68 to 0.92). The displacement of the LSV relative to the prostate was 0.06 mm to the right (CI −0.06 to 0.18, not significant) and 1.13 mm superiorly (CI 1.00 to 1.25). The mean shifts of LSV and RSV were significantly different with the LSV mean shift being superior to the RSV by 0.32 mm (CI 0.20 to 0.44). Interestingly the LSV mean shift was to the right, and the RSV shift was to the left indicating that both shifts were towards the midline, that is they are closer together by 0.44 mm (CI 0.32 to 0.56). (Fig. 1)

### Comparison of fiducials on the left lateral image

**Figure 2:**
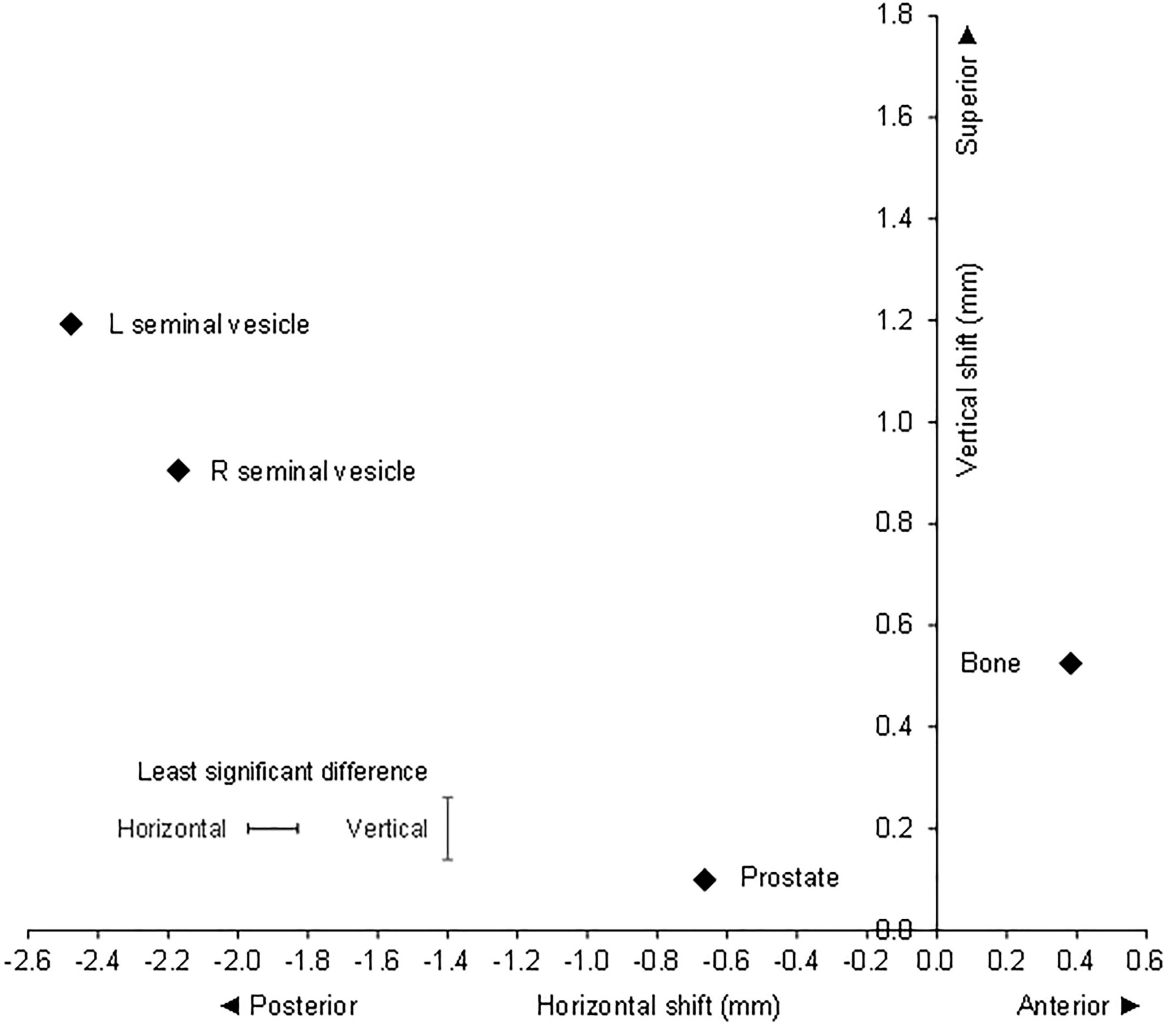
Left lateral image: Mean correctional verification shifts with respect to bone and gold seed fiducial in prostate, right seminal vesicle and left seminal vesicle.

The analysis of variance showed that there were highly significant differences between the fiducials, both in the AP and SI directions (*P* < 0.001, *n* = 9280) (Fig. 2). The prostate and both RSV and LSV shifted posteriorly, and the bone shifted anteriorly compared to the initial setup. Both SV and bone shifted superiorly, with the LSV moving the most.

The mean shifts in mm for each verification method (AP, SI) were prostate (−0.66, 0.10), RSV (−2.17, 0.91), LSV (−2.48, 1.19) and bone (0.38, 0.53). The standard errors of difference between means for the AP and SI directions were 0.073 and 0.063 mm, respectively. The least significant differences (*P* < 0.05) were 0.14 and 0.12 mm for the AP and SI directions, respectively. All differences were significant (*P* < 0.05), so no adjustments were required for the LSD_0.05_.

The RSV and LSV correctional shifts in the AP and SI directions were significantly greater than the prostate. The displacement of the RSV relative to the prostate was 1.51 mm (CI 1.36 to 1.65) posteriorly and 0.81 mm (CI 0.68 to 0.93) superiorly. The displacement of the LSV relative to the prostate was 1.81 mm (CI 1.67 to 1.96) posteriorly and 1.09 mm (CI 0.97 to 1.22) superiorly. The mean shifts of LSV and RSV were significantly different, the LSV shift being greater than the RSV shift with a displacement of LSV relative to RSV of 0.31 mm (CI 0.16 to 0.45) posteriorly and 0.29 mm (CI 0.16 to 0.41) superiorly (Fig. 2)

### PTV margins

The CTV to PTV margins of the prostate and PrSV were calculated for both verification images for each axis (Table 1). The margins for the SV are larger than for the prostate, except in the case of LR shifts for the RSV. The difference in margins between the RSV and prostate were LR 0.3 mm, AP 4.9 mm and SI 1.8–2.4 mm. The differences between the LSV and prostate margins were LR 2.3 mm, AP 3.9 mm and SI 1.9–2.3 mm.

**Table 1:** PTV margin calculation based on prostate and seminal vesicle shifts.

Matched On	AP image							
	+ Right / − Left				+ Superior / − Inferior			
	Overall Mean	SD mean	RMS SDs	Margin	Overall Mean	SD mean	RMS SDs	Margin
		Systematic Error	Random Error	(mm)		Systematic Error	Random Error	(mm)
	(M)	(Σ)	(σ)		(M)	(Σ)	(σ)	
Prostate	0.21	1.354	2.162	4.90	0.53	1.315	2.900	5.32
R S V	−0.17	1.372	2.553	5.22	1.33	1.963	3.147	7.11
L S V	0.29	2.103	2.706	7.15	1.65	2.109	3.303	7.58
Bone	0.36	1.503	2.165	5.27	0.69	2.137	2.627	7.18

Matched On	Left lateral image							
	+ Anterior /−Posterior				+ Superior /−Inferior			
	Overall Mean	SD mean	RMS SDs	Margin	Overall Mean	SD mean	RMS SDs	Margin
		Systematic Error	Random Error	(mm)		Systematic Error	Random Error	(mm)
	(M)	(Σ)	(σ)		(M)	(Σ)	(σ)	
Prostate	−0.66	1.110	2.843	4.77	0.10	1.450	2.780	5.57
R S V	−2.20	2.899	3.524	9.71	0.91	2.301	3.191	7.99
L S V	−2.48	2.426	3.685	8.65	1.20	2.098	3.244	7.52
Bone	0.38	2.159	2.439	7.10	0.53	2.270	2.533	7.45

Overall mean (M) = mean of the mean shifts for 30 patients = group systematic error, SD mean (Σ) = standard deviation of the mean shifts for 30 patients = systematic error, Root Mean Square (RMS) SDs (σ) = square root of the mean of the squares of the standard deviations for 30 patients = random error, and PTV margin = 2.5Σ + 0.7σ.

## Discussion

This exploratory study specifically assessed the motion of the PrSV, rather than the entire SV. Gold fiducials were used to define the prostate as well as the PrSV to approximate a point to point co‐registration and minimise observer and verification error.

This study found significant displacement of the SV relative to prostate verification using gold fiducials and to bone match. We observed significant movement of the RSV relative to LSV. The LSV showed the greatest displacement. This can be expected as the displacement of the SV is mainly due to rectal filling,^10^, ^12^ which is usually asymmetrical. The calculated margins for both SV were greater than the prostate.

While it is recommended that the PrSV be included in the CTV, studies of SV motion have investigated the entire SV. They have confirmed that the SV move relative to the prostate. The prostate was defined by three fiducials or contoured prostate centroid. The movements, both systematic and random, of the entire SV, were reported to vary from 1.1 to 1.9 mm and 0.4 to 1.4 mm LR, 2.8 to 7.3 mm and 1.2 to 3.1 mm AP, and 2.2 to 3.6 mm and 0.06 to 2.1 mm SI, respectively. The PTV margins ranged from 4.5 mm to 15 mm.^10^, ^11^, ^12^, ^13^, ^15^, ^19^, ^20^, ^21^

The variation in SV displacement measurements is likely due to differences in methodology in defining the prostate and SVs. Additionally, investigations of SV motion have varied in the verification method, patient number and type and number of scans. Most investigations of SV displacement have contoured the entire SV length +/− prostate on multiple CTs. These studies are subject to a greater observer variability as it is difficult to define the PrSV on CT or cone‐beam CT (CBCT). The PrSV is attached to posterior prostate over a varied length and is difficult to differentiate as they have similar Hounsfield unit and grey values. Therefore, defining the start of the SV adjacent the prostate and subsequently, its length and position is subject to observer variation. The SV is more easily defined on MRI as they are hyperintense on T2‐weighted imaging compared to the prostate.

CT contouring studies of prostate and SV have illustrated this observer variation.^22^ A report on the intra‐physician variation for prostate contouring calculated a variation of 0.8, 1.1, 1.5 mm for the posterior, anterior and right–left direction with an inter‐observer standard deviation of 1.5, 1.4 and 2.0 mm in the posterior, anterior and right–left directions.^22^ For the SV, they reported inter‐observer variability of 1.5, 2.8 and 2.3 mm in the posterior, anterior and lateral directions and intra‐observer variability of 1.2, 1.2 and 1.5 mm, respectively. The largest CT inter‐observer variation appears to occur at the prostatic apex.^23^ This ranged from 5.4 to 10.7 mm.^23^ Importantly, this observer variation is not dissimilar to and sometimes greater than the measure of the SV displacements. The observer uncertainty may cloud the precise measurement and direction of the SV motion.

Other investigators used greyscale matching techniques to calculate changes in SV position relative to the prostate. A notable study compared entire SV greyscale registration using CBCT, relative to prostate implanted fiducials.^15^ They noted significant systematic and random SV displacements of 1.6 mm and 2.0 mm in LR direction and 2.8 mm and 3.1 mm in the AP direction, respectively. They did not find any difference between the RSV and LSV. These measurements are useful for entire SV as they are not subject to contouring errors and relate to a verification method that is used clinically. However, the measurements may be subject to registration errors, especially when dealing with issues such as seminal vesicle deformation.

Another interesting study quantified the PTV margins required to provide the adequate dosimetric margin of the entire SV versus the proximal 1 cm SV.^24^ Twenty patients had three CT scans, and the contoured SVs were related to three intra‐prostatic fiducials. They illustrated that the SVs move differentially from the prostate with a greater variation and distance. To ensure 95% coverage for 90% of patients, a margin of 8 mm and 5 mm was required for the entire SV and PrSV, respectively. The PrSV margin was the same as the prostate margin. Conversely, our study found that the margin for the PrSV was greater than that of the prostate. The differences may have related to the different lengths of SV used in this study, that is 1 cm versus 2 cm in our study. Other causes may have related to observer variation with organ delineation and motion assessment technique.

MV imaging was selected for verification as the study protocol, and implementation occurred before and during a rapid departmental transition from MV to KV imaging and then CBCT. KV imaging affords better tissue contrast and CBCT provides volumetric imaging and rotational shifts. However, to maintain consistency with the initial patients, it was decided to continue with MV as the gold fiducials were well visualised, only translational shifts were collected, and a well‐performed prospective study by Moseley et al. had shown a highly significant correlation of isocentre shifts between MV, KV and CBCT fiducials.^25^ Conversely, Gill et al. attributed a statistically significant smaller setup error distribution with MV portal imaging compared to KV to the better image quality of KV.^26^

The present study minimised observer uncertainty by using gold fiducials, multiple observers and all verification images. However, the impact of SV deformation or seed migration was not assessed. CT quantification of SV deformation analysis may be difficult as the PrSV origin is difficult to differentiate from the posterior prostate. Thus, it would be ambiguous as to whether the deformation, for example shortening or lengthening, was occurring uniformly or differentially along its length. This could add to uncertainties in contouring and registration studies of SV motion, whereas translational shifts of a fiducial defining the PrSV will at least, in part, reflect the deformation in the calculated margins. MRI may provide a more precise analysis of SV motion and deformation.

Fiducials were inserted into PrSV under ultrasound guidance, and position confirmed at CT simulation. CT and CBCT may have detected gross fiducial migration. However, limited migration would be challenging to differentiate from SV displacement or deformation. Reassuringly, most fiducial studies of prostate and other organs have shown only a very small proportion of fiducials migrate during radiotherapy, and the distance is small.^27^, ^28^ Consequently, it is unlikely that fiducial migration would greatly affect the results.

In summary, a gold fiducial inserted into the seminal vesicle defines a fixed anatomical segment of the PrSV. The subsequent shifts reflect motion and deformation that can be followed during the radiotherapy course. The major caveat being possible migration of the fiducial; however, studies of gold fiducials in prostate and other sites have suggested that this infrequent. In comparison contouring matching methodologies are subject to uncertainties in relation to prostate contouring, seminal vesicle contouring or both of approximately one to 10 mm. These methodologies can be affected by deformation and rotation as the different segments of the PrSV may be contoured even if it is the same length

The clinical scenario of gross SVI, either clinically or MRI, was not addressed. The radiotherapy volume should cover the extent of SVI on MRI and consideration to treat the entire SV would seem appropriate with gross SVI. Margins may need to be greater to cover the entire SV, although a recent study has shown that grossly involved SV is less mobile.^29^

Many of the caveats of this and previous studies of inter‐fraction displacement, including migration, deformation and intra‐fraction motion would be addressed with MRI volumetric imaging that includes cine (4D) studies.^30^ and possibly MRI fiducials. An elegant study of intra‐fraction SV displacements that contoured the entire MRI on cine‐MRI showed that the SV centroid moved significantly more than the prostate in the superior–inferior direction but not in the anterior–posterior or left–right directions.^30^ The displacement increased with time until 10 minutes, after which it plateaued. They concluded that the SV required larger margins in the superior–inferior directions. Furthermore, more sophisticated solutions to PrSV inter‐fraction and intra‐fraction motion could be investigated with adaptive radiotherapy techniques to account for both position and shape.

## Conclusion

The study confirms that the PrSV displacement is greater than the prostate. While margin expansion is departmental specific, this study has illustrated that larger inter‐fraction margins for PrSV should be considered, but careful attention is required regarding the organs at risk, notably the rectum, especially when considering hypo‐fractionated radiotherapy.

## Financial Support/ Funding

No financial support/ funding for this study.

## Conflict of Interest

The author declares no conflict of interest.

## Data Availability

Research data are stored in an institutional repository and will be shared upon request to the corresponding author
